# Public Health Importance of Emporiatrics: A Review

**DOI:** 10.7759/cureus.36343

**Published:** 2023-03-19

**Authors:** Ashok M Mehendale, Lokesh Vaishnav, Shiv H Joshi, Abhishek Joshi

**Affiliations:** 1 Community Medicine, Jawaharlal Nehru Medical College, Datta Meghe Institute of Higher Education and Research, Wardha, IND

**Keywords:** pre-travel consultation, emporiatrics, travel advice, travel health, mental health issues, travel guidelines, vaccines, travel medicine

## Abstract

People travel all around the world to explore, trade, sojourn, etc. Millions of individuals cross national and international borders. Travel medicine services are offered by general practitioners, specialized travel clinics, or immunization centers. Epidemiology, illness prevention, and travel-related self-treatment are all included in the interdisciplinary field of travel medicine. The main objective is to keep travelers alive and in good health, by reducing the effects of illness and accidents through preventative measures and self-care. The danger to a traveler's health and well-being must be understood, and the travel medicine practitioner's job is to help their patient or client recognize and manage those risks. The absence of any disease or symptom does not always indicate good health. Chronic illness sufferers, including those with cancer, diabetes, and hypertension, can maintain a reasonable level of health and mobility. Travel medicine is a rapidly developing, extremely dynamic, multidisciplinary field that calls for knowledge of a range of travel-related illnesses as well as current information on the global epidemiology of infectious and non-infectious health risks, immunization laws and requirements around the world, and the shifting trends in drug-resistant infections. Pre-travel consultation aims to reduce the traveler's risk of disease and harm while on the road through preventive counseling, education, recommended drugs, and essential vaccines. Specialized medical guidance can help reduce the potential health risks of travel. Emporiatrics is not only used for traveling advice or things to be done during the period of the journey but it also creates room in implementing the interdisciplinary subject with new methods or development of new policies, technologies, and various programs to reduce unnecessary problems of the travelers, which will boost tourism.

## Introduction and background

Frequently seen as a part of infectious/tropical medicine, public health, and general practice, travel medicine is rapidly being recognized as a separate clinical and academic multidisciplinary specialty [1,2].

The science of travel medicine

The term “emporiatrics” is given to the field of medical science dealing with travelers' health. In Greek “emperos” means traveler, and “iatrics” is medicine. The field of travel medicine has enhanced as a result of the massive rise in international travel made possible by the explosive growth of tourism, quick air travel around the globe, the building of transcontinental roads and railroads, massive cruise ships, and travel to and exploration of previously inaccessible or remote regions [1,3,4]. This area of medicine focuses on traveler health and addresses illness prevention, immunization, pre-travel counseling, and the epidemiology of health hazards to international travelers. According to the United Nations World Tourism Organization (UNWTO), in the first quarter of 2022, there were an expected 117 million foreign visitors compared to 41 million in the first quarter of 2021, a rise of 182% year over year [5].

**Figure 1:**
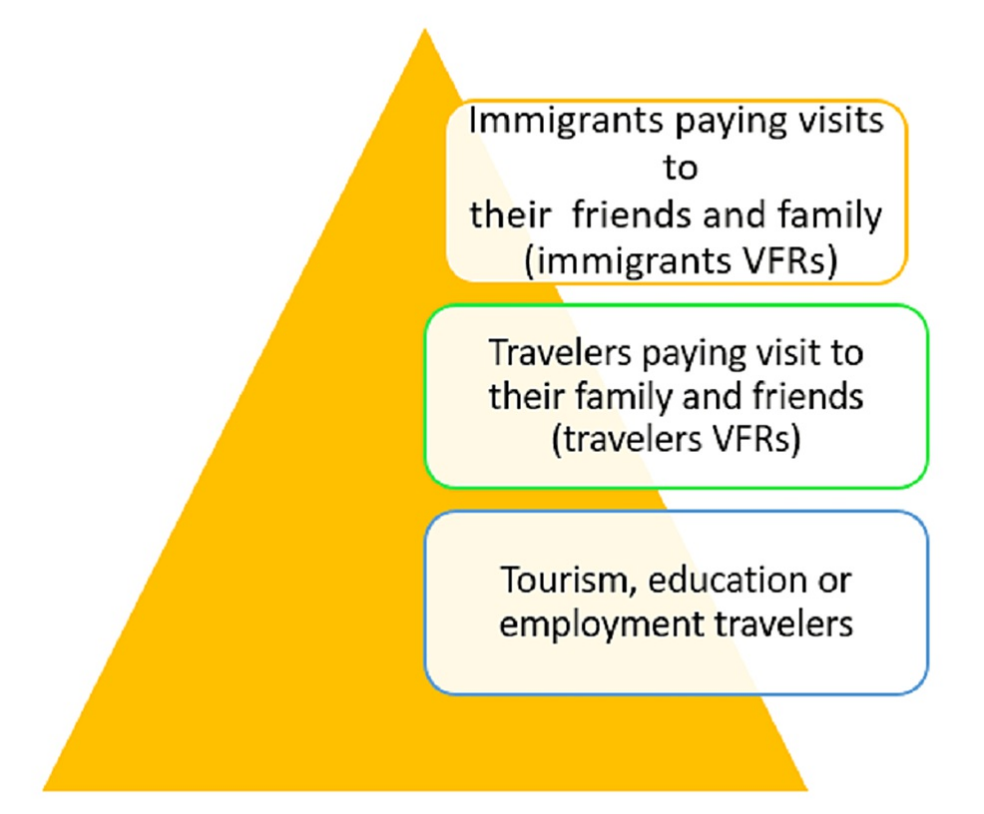
Classification of travelers Immigrant VFRs consist of immigrants who are visiting friends and family back in their place of origin. These travelers contact more travel-related illnesses, require hospitalization for these illnesses more frequently, and seek less pre-travel medical advice; Traveler VFRs consist of travelers who are not originally immigrants but are going back for the visit; Tourism, education, or employment travelers comprise people who travel for business, pleasure, education, or employment. These travelers frequently seek more pre-trip medical advice, experience fewer travel-related illnesses, and infrequently need hospitalization following their journey. VFRs: visiting friends and relatives Image source: Author

Travel medicine is defined as the field of medicine concerned with the promotion of health for the peoples, cultures, and environment of regions being visited in addition to the prevention of disease or other adverse health outcomes in the international traveler. It focuses primarily on pre-travel preventive care [6]. Understanding travel-related illnesses, epidemiology, immunization regulations, and drug-resistant infectious diseases are vital for practicing travel medicine [7]. Figure 1 depicts the classifications of the travelers visiting their family and friends as immigrants and travelers [8].

## Review

Methodology

In September 2022, we conducted a thorough search using PubMed Central using keywords and medical subject headings (MeSH) such as “emporiatrics” and “travel medicine” (((emporiatrics [title/abstract]) or (emporiatrics [MeSH terms])) and (travel medicine [title/abstract])) or (travel medicine [MeSH terms]). Additionally, we searched through the bibliographies of pertinent papers to find essential references. Updates to the search were made in January 2023. Based on the title and abstract first, and subsequently, the full texts, one reviewer independently evaluated the retrieved papers against the inclusion criteria. Discussions were held to settle disagreements.

Global tourism

Globally, tourism has dominated the process of achieving sustainable development goals (SDGs) by the year 2030 and is considered to be a target in three out of 17 SDGs (target 8.9, 12.b, 14.7) [9]. For approximately four to six weeks prior to departure, WHO suggests that travelers consult with a physician specializing in travel medicine. A major part of travel medicine is pre-travel advice for older travelers, individuals with complex medical conditions, and the substantial number of ethnic tourists who visit their nation of origin to see friends and family [10]. Up to 8% of tourists will seek medical attention for these occurrences since the disease can happen anywhere, at any time [11,12].

Coronavirus disease 2019 (COVID-19) and traveling

Following the industrial revolution, a wave of globalization propelled countries to sign free trade agreements [13]. People started migrating and moving more as a result of globalization, which was made possible by science and technology. Microbes also moved beyond where man did. Public health has become an international problem as a result of globalization, urbanization, and population growth because of the transnational spread of newly emerging infections, the resurgence of established infections, and zoonoses [14,15]. If the COVID-19 epidemic is not swiftly eradicated worldwide, its psychological, societal, and economic effects will be more severe. The establishment of short-term travel restrictions by the nations sought to quell the widespread panic caused by the COVID-19 outbreak [16]. To understand more qualitative assessments in-depth interviews are required.

Traveling and physicians' concerns

Healthcare practitioners must pay particular attention to the patterns of infectious illnesses since they vary by geographical location, population, and variances in climate [17,18]. It is strongly encouraged that pre-travel care be given by qualified individuals who carry a certificate of competence in the field and have frequent experience advising tourists with a variety of complex health issues, destinations, and itineraries (such as that offered by the International Society of Travel Medicine) [19]. One of the greatest ways to minimize the spread of antibiotic resistance is to advise tourists, on infection prevention and pre-travel vaccinations.

The following are crucial components of travel medical practice: knowledge, training, and experience in the field of the provider, risk assessment of the traveler, advice on the prevention and management of travel-related diseases (both infectious and non-infectious), capability to counsel travelers of all ages and with various medical conditions, ability to administer vaccines and identify key syndromes in returnee travelers. To be aware of the risks when treating patients who have a history of traveling abroad, clinicians and private medical professionals should become familiar with travel medicine and the illnesses that may impact them [20].

Family physicians' and community pharmacists' responsibilities

Doctors who treat families in their community and local pharmacists are typically the first points of contact for people contemplating overseas travel; they are critical in identifying at-risk travelers and emphasizing the importance of receiving a pre-travel consultation [19]. According to the Committee to Advise on Tropical Medicine and Travel and the International Society of Travel Medicine, all high-risk passengers should be directed toward travel medicine professionals who have experience providing specialized care and attending to these specific needs [21]. All the patients who come in should be often questioned about their plans to travel overseas, particularly to a developing country [22]. Pharmacists need to be concerned about the traveler's multidimensional travel risk against antibiotic use when traveling abroad [23].

Basics of pre-travel consultation

**Figure 2:**
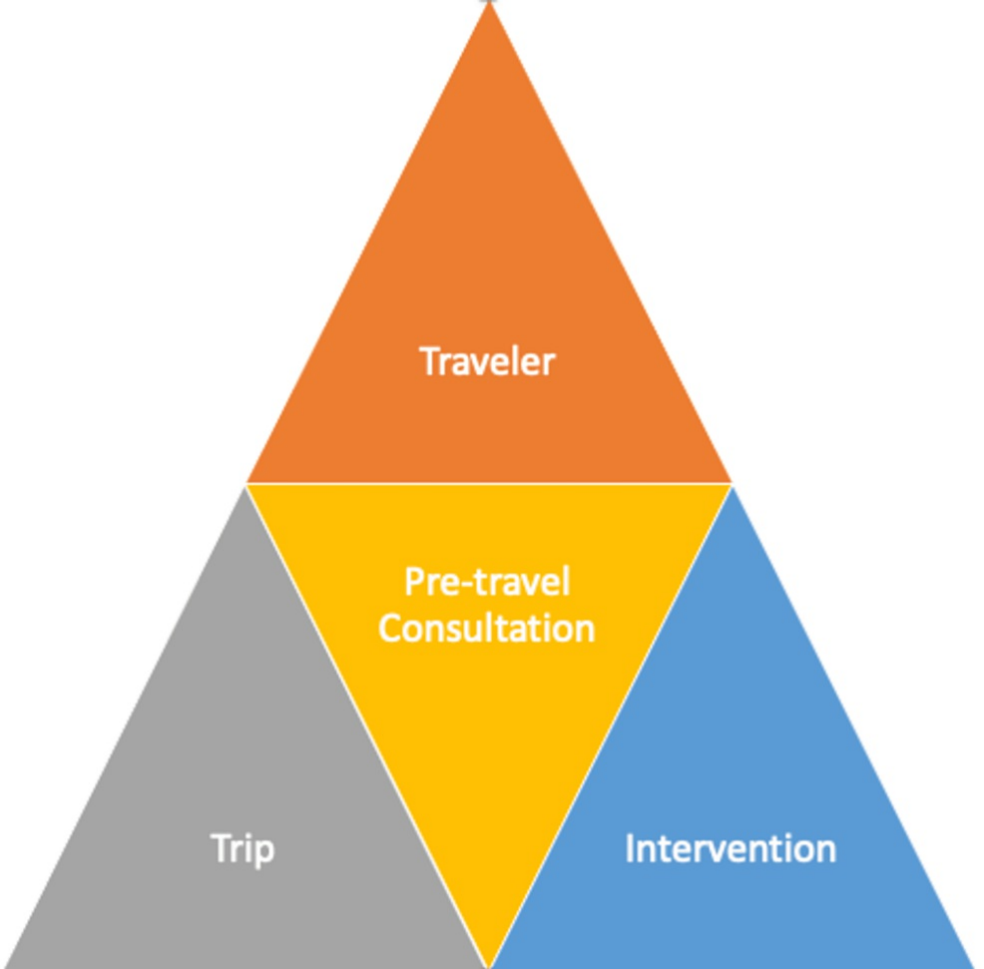
Pre-travel consultation triad Image source: Author

Preventive counseling, education, prescribed medications, and necessary immunizations are all part of the pre-travel consultation's objective of lowering the traveler's risk of disease and damage while on the road [12,24]. Figure 2 shows that pre-travel consultation comprises a triad of the traveler who is on the verge of traveling, a trip that is determined by the traveler, and intervention taken to make the travel successful. Before traveling, travelers should be informed of the risk of illness in the country or countries they intend to visit, as well as the precautions they need to take to keep healthy. No single immunization schedule is appropriate for all travelers. Each plan needs to be customized based on the traveler’s prior immunizations, the nations they intend to visit, the nature and length of their trip, and the amount of time they have before leaving.

Similarly, before traveling, a doctor's appointment is a good time to review the immunization records of the patients and administer any routine vaccines that have been missed in addition to the travel-related vaccines that are required [25]. In the case of low-risk diseases, education regarding risk avoidance may be a more cost-effective strategy than vaccination. Creating a connection with people or a specialty facility that may offer such treatment if travelers become unwell upon their return has become an important aspect of providing pre-travel care [26].

In order to increase patient knowledge of the hazards associated with travel, the pre-travel clinical evaluation includes risk identification, classification, and counseling. Table 1 demonstrates the evaluation, which is best performed four to six weeks before departure, taking into account the travel destination itinerary, travel history, pre-existing medical concerns, immunization history, behavioral risk factors, and biological health threats [27].

**Table 1:** Pre-travel concerns

Sr. No.	Topics	Remarks
1	Itinerary, mode of transportation, and length of travel	Scheduled events related to planned travel.
2	Pre-existing patient health conditions and treatment history	Age, persistent medical conditions, medications, immunological status, and pregnancy are just a few variables that may impact the epidemiologic risk of some destination-related diseases.
3	History of immunization	Patients seek pretravel medical evaluations, for this reason, the most frequently. All recommended regimens for routine childhood vaccinations should be reviewed. Immunizations may be mandatory (for example, yellow fever) or advise based on personal risk depending on the destination.
4	Avoidance of animal and insect	Avoiding stray animals, dressing appropriately, limiting skin exposure, using insecticide-infused bed nets, and using insect repellents with at least 30% N, N-diethyl-m-toluamide (DEET) are all effective ways to prevent animal or vector-borne infections.
5	Precautions for food and water	By staying away from foods that can't be boiled, peeled, or completely prepared, the gastrointestinal diarrheal disease can be prevented. Eliminating ice in beverages, brushing your teeth with tap water, and only drinking purified water are further protective measures (e.g., bottled, boiled, iodinated, microfiltered, and carbonated).
6	Concerns for environment	Remind passengers to take precautions against accidents, high and cold temperatures, sun exposure, and swimming-related dangers. Seat belts, helmets, traveling in pairs or small groups, and drinking enough of water are further protective measures.

Disease burden related to travelers

The diseases related to traveling are the diseases that come directly from traveling to a specific location, whether inside a country or a person traveling internationally [28].

Malaria 

Malaria is globally prevalent, with an incubation period of 9-14 days. Cycles of fever every second and third day with sweating, myalgias, a severe headache, and fatigue are general symptoms. Whereas, acute respiratory distress syndrome, coma, altered mental status, kidney failure, and/or death are symptoms of severe illness. *Plasmodium falciparum* infection is considered a medical emergency that can result in an actual, life-threatening condition, and treatment should begin as soon as possible. The type of malaria, the chance of medication resistance (depending on where the illness was contracted), the patient's age, whether she is pregnant, and the severity of the infection all affect the available treatments. Prevention is done via chemoprophylaxis: artemisinin and proguanil, 250mg atovaquone + 100mg proguanil per oral (PO) daily, starting two days pre-exposure and continuing for seven days post-exposure; chloroquine 500mg salt (300mg base) PO weekly, starting two weeks pre-exposure and continuing for four weeks post-exposure; doxycycline 100mg PO daily starting two days pre-exposure and continuing four weeks post-exposure; mefloquine 250mg salt (228mg base) PO weekly, starting two to three weeks pre-exposure and continuing four weeks post-exposure [27].

Dengue 

Dengue** **is geographically prevalent in the tropics and subtropics and the incubation period is 4-10 days. Treatment: supportive care. Prevention: appropriate protective measures [27].

Chikungunya

Chikungunya depicts the excruciating agony that someone with arthritis experiences, and demographically the rainy seasons are when chikungunya is most prevalent in the tropics and subtropics. The incubation period is 1-12 days. Symptoms include acute febrile illness with a temperature of 39 degrees Celsius, which means 102 degrees Fahrenheit. There are some instances of headache, conjunctivitis, nausea, and/or vomiting, together with a maculopapular rash. Treatment: supportive care. Prevention: appropriate protective measure [27].

Zika

Zika was discovered in the Zika forest of Uganda and is linked with occasional outbreaks in the tropics and subtropics, namely in Latin America, the Caribbean, Southeast Asia, and Africa. The incubation period is 3-12 days. The *Aedes aegypti* and *Aedes albopictus* mosquitoes are the primary carriers. Symptoms are described as a mild, febrile illness with a maculopapular rash, arthralgias, and conjunctivitis with a mild headache and myalgias. For pregnant women, there are chances of fetal microcephaly and spontaneous abortions. Treatment: Supportive care. Prevention: Appropriate clothing, indoor insecticides, and repellents [27].

Leptospirosis

Leptospirosis is a zoonotic disease. It’s an obligately aerobic, Gram-negative spirochetes with worldwide distribution. Travelers are more in danger while visiting the tropics and subtropics during the rainy season. The incubation period is 2-30 days. Transmission happens through the surface waters tainted with the infected urine of animals, notably dogs. In patients who progress to severe disease, the illness can be biphasic, with a temporary decrease in fever between phases. The acute, septicemic phase presents as an acute febrile illness. In severe cases, manifestations like jaundice, renal failure, and bleeding are seen. Treatment for severe infection: penicillin G 1.5mu intravenously every six hours for seven days; treatment for mild infection and non-pregnant females: doxycycline 100mg orally every 12 hours for seven days; treatment for mild infection, pregnant female, and child under 12 years: amoxicillin 500mg orally every eight hours for seven days. Prevention: frequent handwashing and avoiding contaminated food, drink, and potentially diseased animals or their bodily fluids [27].

Travelers’ Diarrhea

Travelers’ diarrhea is self-treatable. It is defined as the passage of unformed stools 24 hours after travel, plus accompanying symptoms like nausea, vomiting, stomach pains, fever, tenesmus, or bloody stool. The countries with the highest risk of infection include Central and South America, Africa, Asia, the Middle East, and Mexico. Bouts of diarrhea happen during the first 7-10 days just after the traveler returns home. *Aeromonas* and *Plesiomonas* species, enterotoxigenic *Bacteroides fragilis* group species, *Campylobacter jejuni*, *Shigella* and *Salmonella* species, and enterotoxigenic *Escherichia* *coli* species can also cause infections, while enterotoxigenic *Escherichia coli *is the most common bacterial cause. The fecal-oral transmission pathway has a 6-72 hour incubation time for bacterial and viral diseases. The incubation time for parasite infections might range from one to two weeks. Treatment is the cornerstone of therapy with sufficient and timely rehydration with antibiotic medication: azithromycin 1,000mg as a single dose, azithromycin 500mg once daily for three days, ciprofloxacin 500mg twice daily for three days, or rifaximin 200mg daily for three days. For the parasitic regimen: metronidazole 250mg three times daily for five days. Keeping away from the ice, shellfish, and sauces on restaurant tables, drinking from straw-equipped bottles, and avoiding salads and buffets where food may have been left out of the refrigerator for several hours are additional general advice. Everywhere feasible, travelers are advised to consume bottled water [28-30].

Pre-travel health checklist for expectant women 

Immunity is to be examined for contagious illnesses, including hepatitis A and B, varicella, measles, and pertussis; update about the vaccines as necessary. Table 2 gives the critical points as contraindications for females traveling during pregnancy [29].

**Table 2:** Contraindications of travel during pregnancy

Absolute Contraindications	Relative Contraindications
Abruptio placentae; Active labor; Incompetent cervix; Premature labor; Premature rupture of membranes; Suspected ectopic pregnancy; Threatened abortion, vaginal bleeding; Toxaemia, past or present	Abnormal presentation; Fetal growth restriction; History of infertility; History of miscarriage or ectopic pregnancy; Maternal age <15 or >35 years; Multiple gestations; Placenta previa or other placental abnormality

Guidelines and Required Documentation

(a) Travel health insurance and medical evacuation coverage (research specific coverage information and limitations for pregnancy-related health issues) [29]; (b) Verification of the cruise lines and airlines' policies regarding pregnant guests; (c) Confirmation letter for the due date and travel eligibility; (d) Copy of medical records

Preparing at the Destination for Obstetric Care

(a) Medical insurance coverage; (b) Necessary arrangements for obstetric care at the destination

Warning Signs and Symptoms That Need Medical Attention

(a) Pelvic or abdominal pain; (b) Bleeding; (c) Rupture of membranes; (d) Contractions or preterm labor; (e) Symptoms of pre-eclampsia (unusual swelling, severe headaches, nausea and vomiting, vision changes); (f) Vomiting, diarrhea, and dehydration (g) Signs and symptoms of potential deep vein thrombosis or pulmonary embolism (unusual swelling of leg with pain in calf or thigh, unusual shortness of breath) [31]

Water disinfection for travelers

One of the risks to travelers is posed by water-borne diseases. Foreign travelers traveling to unclean and unsanitary places and wilderness that rely on surface water are at risk of developing a waterborne disease. The danger of waterborne infection is greater in areas with untreated surface or well water and poor sanitary infrastructure. Small infectious doses of microorganisms (including *Giardia, Cryptosporidium, Shigella, Escherichia coli *O157:H7, and norovirus) can even make people sick when they accidentally consume water while enjoying recreational activities [30]. Bottled water is one of the safest options for travelers. All expatriates and long-term tourists should ensure the consumption of clean drinking water and follow these straightforward techniques: boiling, filtration, and chemical disinfection with substances such as halogen compounds (chlorine, iodine resins, salt electrolytes), chlorine dioxide, ultraviolet light, solar irradiation and heating, silver and other products [32].

Methods of food and water consumption for travelers 

(a) Consumption of tap water, washing raw foods in it, or using it to make ice should be avoided; (b) Open buffets, uncooked meat, seafood, and unpasteurized dairy products should be avoided; (c) Only boiled, treated, or bottled water should be used for consumption; (d) Only food that is boiling hot and fully cooked should be consumed; (e) Only eateries and restaurants with a stellar safety reputation should be visited; (f) Personal precautions should be taken to avoid contracting diseases from insects [29,32]

Overall health travel prevention

The popularity of tropical destinations among tourists has increased the number of patients who seek medical advice on the health risks inherent in hot countries and the health protection measures to be taken there, especially where sanitation is insufficient. The likelihood of contracting a disease while traveling overseas is influenced by a number of variables, such as the traveler's overall health, health prevention measures taken before or during the trip (vaccinations, antimalarial chemoprophylaxis, health precautions during air, land, and sea travel, proper acclimatization, prevention of heat injuries, protection against local flora and fauna, personal hygiene, water, food, and feeding hygiene), as well as the prevalence of the disease. [33].

Prevention against insect bites

Travelers should get medical advice on practical precautions before entering regions where vector-borne diseases are endemic. Travelers are advised to wear insect repellents with 30-50% N,N-diethyl-meta-toluamide (DEET) or 20-30% picaridin to avoid being bitten by insects. Long-sleeved shirts or tops, long pants, and other clothing covering as much of the body as possible are recommended for travelers to tropical areas. They should also avoid being outside from dusk to sunrise [34].

Vaccines for travelers

Vaccinations must be adjusted to the traveler’s individual immunization history, the destinations, the type and length of the trip, and the amount of time until departure. To allow for the completion of the necessary immunization regimens, the healthcare provider should ideally be notified three months before departure [6,35]. Table 3 notifies WHO-provided vaccine guidelines for travelers traveling to different countries [36,37]. The history of vaccine policy, political and cultural challenges, and the importance of the vaccine mandate are crucial considerations for policymakers. Vaccination laws may vary from one nation to another depending on their motivation, layout, target audience, and enforcement. The onset or eradication of epidemics, major related morbidity and mortality, lack of treatment, impact on tourism, and the introduction of new vaccinations are some factors that may affect vaccination policies and regulations for tourists [38].

**Table 3:** Vaccination guidelines for travelers (WHO) WHO: World Health Organization; ITH: International Travel and Health Adapted from: Stringer et al., 2002 [39]

Categories of Vaccine	Vaccine Description	Example
Routine vaccines for traveling	Several national childhood vaccination programs include these vaccines. However, a pre-travel consultation is an excellent chance for medical professionals to examine the level of immunization for newborns, kids, teenagers, and adults and make sure they have had all necessary shots in accordance with their own country's schedule.	Diphtheria, tetanus and pertussis, hepatitis B, hemophilus influenza type B, human papillomavirus, influenza (seasonal), measles, mumps, and rubella pneumococcal, polio, rotavirus, tuberculosis, varicella
Required vaccines for certain countries	Vaccines are advised to guard against illnesses that are indigenous to the place of origin or the country of travel. They are designed to safeguard travelers and stop the spread of illness both inside and between nations.	Hepatitis A and E, Japanese encephalitis, meningococcal, polio (adult booster dose), typhoid fever, yellow fever, rabies, encephalitis
Recommended vaccines by some nations	Some nations demand vaccination documentation from visitors who want to enter or leave the country. Consult the WHO's ITH website's country list for further details.	Polio vaccine, yellow fever vaccination for travelers entering and leaving regions or countries at risk for the disease, meningococcal vaccine.

Visitors to regions with high levels of anti-microbial resistance (AMR) should be upgraded on their vaccinations, knowledge about alternatives to using antibiotics for treating and preventing travelers' diarrhea, and trained in safe sexual behavior. Investments are needed in low and middle-income nations' healthcare systems to increase access to clean water, sanitary facilities, and vaccines and decrease the spread of resistant strains [40].

Infections transmitted sexually and by blood

The prevention of sexually transmitted diseases (STDs) and blood-borne illnesses should be given top priority by travel clinic services (e.g., HIV and hepatitis B). Some factors that are linked to STDs include male sex, being single, being under 20 years old, traveling alone, having had more than two sexual partners in the previous two years, being a casual user of illicit substances, or being an alcoholic, as this cadre has higher engagement in sexual intercourse. Visits made specifically for the purpose of procuring sex are known as “sexual tourism”. Such travelers are more likely to contract diseases like AIDS, HIV, monkeypox, COVID-19, etc. due to sexual encounters [41,42]. The primary prevention approach is centered on suggestions for safer sex behaviors, such as restricting the number of new partners, wearing condoms regularly and correctly during sexual activity, and minimizing alcohol and/or illegal drug usage.

Care after traveling

Systemic febrile illness, diarrheal disease, and dermatological disorders are the most typical clinical signs of travel. All suspected incidences of sickness among visitors returning home should be thoroughly and historically documented. Pre-travel vaccinations, travel-related chemoprophylaxis and adherence (e.g., malaria), recent illnesses, and antimicrobial medication should all be covered in the history. The history should contain the person's risk factors. A thorough physical examination is necessary, but the proper testing and treatment should be guided by clinical and epidemiologic concerns connected to recent travel [43]. Table 4 shows the most significant patient education components for immunocompromised travelers, which are crucial for such patients [35].

**Table 4:** Key patient education points for immunocompromised travelers

Sr. No.	Key Points
1	Create a strategy in case you get sick while traveling (To employ embassy resources and medical evacuation insurance; a clinic or hospital that could treat an immunocompromised host.)
2	In case of flight delays, bring extra prescriptions, and make sure they are labeled.
3	Avoid using drugs that you buy at your destination because they could interfere with other medications or be subpar, fake, mislabelled, or counterfeit medical products.
4	Increased risk of contracting multidrug-resistant bacteria when traveling and later; mention such travel to clinicians if you become ill.
5	Due to the significantly increased risk of photosensitivity from medication, immunocompromised hosts should use extreme caution while using sun protection.
6	Stringent food and water safety measures. It could be helpful to use antibacterial hand wipes or an alcohol-based hand sanitizer with at least 60% alcohol.
7	To carry a travel health kit.

Post-travel mental health issues

Traumatic experiences that a traveler may have experienced can lead to acute stress disorder (ASD), and post-traumatic stress disorder (PTSD), and travel health experts may be in a unique position to inquire about these experiences of PTSD.

The following points are to be remembered: (a) Reliving the experience can involve recurrence, bothersome memories, traumatic dreams of the event, and the sensation that it is re-occurring; (b) Avoiding ideas, emotions, behaviors, locations, or people that trigger recollections of the event is one of the signs of avoidance; (c) Reduced interest in activities, an inability to feel positive emotions, or difficulty recalling essential facts about the event are just a few examples of changes in mood or cognition related to the event; (d) Arousal symptoms include trouble falling asleep or staying awake, impatience, or an excessive startle reaction [44,45].

Social media effect on travel medicine 

Social media now encompasses a wide range of internet-based websites and technologies, where information and ideas are created, shared, and traded in virtual communities and networks. Information transmission involves cautious application because it might not always produce the desired result. It also needs to be checked for quality, accuracy, and dependability while maintaining confidentiality and privacy. Websites or platforms like Facebook (Meta Platforms, Inc., Menlo Park, California, United States), Twitter (Twitter, Inc., San Francisco, California, United States), Wikipedia (Wikimedia Foundation, San Francisco, California, United States), and YouTube (Google LLC, Mountain View, California, United States) target the population at a very low cost in view to provide innovative ideas for traveling [46].

Travel apps and ethical considerations

Traveling is a common global phenomenon that greatly impacts expenditure, employment, and health, whether it is for pleasure, business, or VFR [47]. In order to prevent and treat infections that can occur when traveling, travel medication is crucial. Vaccinations, prophylaxis, travel safety information, insect bite prevention, and other preventative measures are important for sustaining travelers’ health [34]. The growing usage of smartphones and improvements in the quality of mobile health technologies have made it easier and more reliable to monitor traveler health behavior as well as encountered threats [48]. WHO and an ambitious new project called Illness Tracking in Travelers (ITIT) are working together to gather information on traveler illnesses in order to facilitate quick public health responses [49]. Many travel medicine applications lack accurate and evidence-based content, are out of date or were not created with the input of medical professionals [50]. Updated regulatory systems are needed to assist researchers and developers in making moral decisions in the rapidly changing world of digital health and health apps.

Future of travel medicine after adapting to the COVID-19 pandemic

Innovation has accelerated because of the COVID-19 pandemic, and travel medicine will need to continue to use it in the future. Weekly updates and improvements were made to travel advice and destination requirements from organizations like the International Air Transport Association, UNWTO, and the CDC Travelers’ Health Branch [51,52]. Estimates on a variety of COVID-19 components have been produced by the field of disease modeling, which has significantly expanded. Using an immersive virtual reality platform, leading travel medicine educational programs successfully transitioned to give high-quality online webinars and courses, online exams, and an international conference [53]. New platforms should be utilized by travel medicine in the future for clinical care, research, and education [54-56].

Discussion

The medical community, the travelers themselves, travel organizations, airline and shipping firms, and host governments must meet the health needs of travelers traveling quickly across countries and continents [57]. The emergence of COVID-19 has raised public awareness of the negative effects of inadequate resilience and pandemic preparedness. But the effects of pandemics can be exacerbated in some developing nations where there is a lack of access to clean water and sanitation, including hand washing. Antibiotics must be used cautiously for treating traveler's diarrhea due to their uncertain efficacy in comparison to supportive therapy and the continuous global spread of AMR [58]. Specialist medical guidance can diminish the health consequences of travel [59].

## Conclusions

Emporiatrics is crucial in preventing traveler problems as they make their way to their final destination. Risk avoidance education may be a more cost-effective approach than vaccination in the event of low-risk diseases. More travel-related accidents have been prevented as a result of the advancements made in this field. Travelers' health risks are addressed by effective public health measures like preventive medicine and vaccination against diseases of concern. The way people travel will be completely transformed by new techniques and technologies that guarantee their health and safety. VFRs and concerns about travel following the pandemic have significantly impacted people's perceptions and information across the globe. Currently, very little is known about the word emporiatrics but it creates a more productive system for preventing traveler-related diseases.
